# Modeling and comparing minimum miscibility pressure using multiple mixing cells for injection of enriched gases with Naphta, LPG, and NGL

**DOI:** 10.1038/s41598-023-31018-8

**Published:** 2023-03-08

**Authors:** Mohammad Amin Rashidi, Mohammad Reza Khademahmadi, Yousef Kazemzadeh, Masoud Riazi

**Affiliations:** 1 Petroleum Engineering, Civil and Environment Engineering Department, University of Alberta, Edmonton, Canada; 2 Enhanced Oil Recovery Research Center, School of Chemical and Petroleum Engineering, Shiraz University, Shiraz, Iran; 3 Department of Petroleum Engineering, Faculty of Petroleum, Gas, and Petrochemical Engineering, Persian Gulf University, Bushehr, Iran

**Keywords:** Engineering, Chemical engineering

## Abstract

The increase in oil production from hydrocarbon reservoirs has always been of interest due to the increase in global oil consumption. One of the effective and useful methods for enhancing oil recovery from hydrocarbon reservoirs is gas injection. Injectable gas can be injected into two modes, miscible and immiscible. However, to inject more efficiently, different factors, including Minimum Miscibility Pressure (MMP) in the gas near-miscible injection mode, should be investigated and determined. In order to investigate the minimum miscible pressure, different laboratory and simulation methods have been prepared and developed. This method uses the theory of multiple mixing cells to simulate, calculate and compare the minimum miscible pressure in gas injection enriched with Naptha, LPG, and NGL. Also vaporizing and condensing process is also considered in the simulation. The constructed model is presented with a new algorithm. This modeling has been validated and compared with laboratory results. The results showed that dry gas enriched by Naphta due to having more intermediate compounds at lower pressure (16 MPa) is miscible. In addition, dry gas, due to very light compounds, needs higher pressures (20 MPa) than all enriched gases for miscibility. Therefore, Naptha can be a good option for injecting rich gas into oil reservoirs to enrich gas.

## Introduction

Measuring the miscibility pressure with appropriate accuracy and appropriate combination for injection in order to carry out increased EOR projects is very important because the pressure and composition used directly affect the project economy^1–3^. Therefore, providing a suitable and easy method for calculating minimum miscibility pressure for designing EOR projects is important and efficient^4–17^. Various laboratory methods have been introduced to measure minimum miscibility pressure including slim tube, rising bubble, and vanishing interfacial tension. One of the most promising methods is microfluidics which is recently have applied in the oil nd gas industry. This method has been applied to determine gas oil ratio, wax appearance temperature, and asphaltene deposition. Also, micro models have been implemented to simulate the pore scale and displacement processes in oil and gas reservoirs. Ungar et al.^18^ developed a novel microfluidic approach that can be applied at high temperature and high pressure condition to reproduce reservoir condition. Also a homogenous pore structured used to simulate the pore geometry of slim tube. They captured the oil displacement in micro model at different pressure. The results showed that remaining oil after specific pressure was negligible which represent MMP pressure^18^.

One of the suitable methods for measuring MMP is multiple mixing cells. Calculating the MMP by a mixing cell approach^19–21^ uses an imaginary fluid cell and an equation of state to forecast the MMP (or minimum miscibility concentration). These methods are based on the assumption of simplifications that the oil or gas equilibrium node line controls intermingling^22^. A fluid cell is then used to make repeated calls between oil and gas forward or backward to converge to the oil or gas equilibrium node line. The criterion for MMP is the pressure in which the convergent equilibrium node line is converted to the critical tie line (i.e., it reaches zero length). Published articles on single-cell mixing methods differ in terms of minimum completing pressure estimation method. However, the main common stages among them are shown below:

1. Forward contacts: The cell is filled with the main oil at a specific pressure and constant temperature. The initial pressure is assumed to be far from the MMP range.
2. Some gas is added to the cell. The amount of gas should be adequate to achieve the overall oil and gas mixture after calculating the equilibrium in two stages.
3. The equilibrium steps are calculated at a constant condition (temperature and pressure).
4. Gas is mixed with the main oil at an equilibrium state.
5. Steps 3 and 4 are repeated until the equilibrium combination is changed with more contacts, i.e., the equilibrium component converges to the oil equilibrium node line.
6. If the equilibrium node line length reaches zero or the specified tolerance, the calculated pressure is the MMP. Otherwise, the pressure increases and steps 1 to 6 are repeated.

The prominent disadvantage of the single-cell model is its weakness in the estimation of the minimum miscible pressure for condensation/evaporator displacements. As Jensen and Mikkelsen^23^ and Orr^24^ discussed in detail, the deficiency of these methods roots in their basic assumption that a binary system can facilitate the phase behavior of oil and gas. Therefore, only one main equilibrium node line can be found by a cell—the oil equilibrium node line with forwarding contacts and the gas equilibrium node line with backward contacts. If any of these equilibrium node lines controls miscibility, single mixing cell methods can accurately predict the minimum miscible pressure. Otherwise, as is the case in most oil and gas compounds, estimating MMP can be error-free.

The aforementioned methods are simple slim tube simulations^25^ that only perform phase equilibrium calculations, and the flow equation's solution is not considered. Numerous methods have been published for multiple mixing cells. However, an overwhelming majority of those methods are based on the study of Cook and their colleagues^26^. Jaubert et al.^27^ introduced the first approach for measuring MMP based on the Metcalf et al. method^28^. The Jaubert et al. method tries to calculate all contributed mechanisms, including condensate/evaporator, similar to one-dimensional simulation. This procedure may be influenced by dispersion. To diminish the dispersion effect, recovery factors are determined in 1.2 volumes of injected pores for pressure and the number of fixed cells. The number of cells changes and the computation of the recovery coefficient is repeated at the same pressure. The recovery coefficient diagram in 1/N, where N is the number of cells, presents the predicted recovery factor in zero-dispersion for that pressure. The zero-dispersion recovery factor diagram is measured for multiple pressures; the resulting extrapolation in 97% recovery shows the pressure associated with the MMP. Another study to calculate the MMP was done by Kariman Moghadam et al.^29^ in which, in fact, the calculations related to mixing in multiple cells are initially performed to calculate the amount of recovery factor in different cells, and then by extrapolation of the obtained data in each cell, the amount of 97% of the recovery factor in the cell is estimated. And the maximum pressure in which we reach RF^1.2^_∞_ = 97% is the same as the MMP. The algorithm used to calculate MMP in this work is that 50 cells are filled with tank oil at first and placed at reservoir temperature and at a pressure less than MMP pressure. Then, 1.2 pores (1.2PV) were injected into them, in which case it was as if the volume of injected gas to each cell would be 33% of the volume of that cell. For each cell after gas injection, equilibrium calculations are performed and the amount of gas and liquid phases in each cell is calculated and then the amount of extra injectable gas is transferred to the next cell. The mentioned steps are repeated to the point that the total injectable gas is injected, and the amount of recovery factor for 50 cells is calculated. In the same way, for more cells such as 100 and 200 cells, the repeated steps are repeated, and the recovery factor diagram is plotted in terms of 1/√N, which is N is the number of cells and the linear chart, and by extrapolation, the recovery factor for cell is obtained. After that, the maximum pressure is selected, and the previous steps are repeated, and by drawing the graph of the international recovery factor in terms of pressure and extrapolation to the recovery factor of 97%, the MMP is obtained. Similar to the aforementioned research, Li and Li^21^ followed the same steps at constant pressure until the last cell, and the amount of liquid and gas were calculated. Then, Tie lie length was calculated and plotted versus pressure and extrapolated until its value reached zero. The obtained pressure (at tie line = 0) was minimum miscibility pressure. The method of multiple mixing cells has many drawbacks. These problems are related to the path in which multiple mixing cell methods are obtained from the simplification of hybrid simulators. Therefore, they inherit simulation bugs, including the dispersion effect. First, apart from Joubert et al.'s method, no one has developed a method of calculating the minimum miscible pressure. Instead, in studies, the development of miscibility and interaction of oil-injected gas has been investigated. Second, all methods of multiple-cell mixing are susceptible to dispersion in scientific sources. Because similar to hybrid simulation, they use a fixed number of cells that have a limited volume. They also transfer a certain volume of liquid from one cell to another without solving the flow equation. Therefore, in order to estimate the correct MMP from mixing cells, we need to reduce the dispersion effect. Jaubert et al.^27^ did this by externalizing recovery to an infinite number of cells. Third, the mixing cell method has not used a strong criterion to determine the minimum miscible pressure, which is the length of the main equilibrium node lines. Only the proposed method of Jabuert et al. uses RF^1.2^_∞_ = 97% (RF^1.2^_∞_ is extrapolated as a recovery factor to an infinite number of cells). However, the value of 97% is experimentally determined through observation. Zhao et al. used the length of the equilibrium node line but not estimate the MMP.

In summary, different methods of mixing cells in scientific resources have been simulated in simple terms, and scientific researchers have further studied the development of miscibility rather than calculating MMP; all of these methods used cells with limited volume, and therefore, like simulations, their results for dispersion should be modified. Most proposed mixing cell methods cannot estimate the MMP for condensate/evaporator displacement. Furthermore, in the multiple-cell mixing method, estimating minimum miscibility pressure is not based on a strong criterion and is not well described in scientific sources. For example, in the Joubert et al. method, choosing the next pressure increase requires other calculations. In this study, the MMP is calculated using a new algorithm. In this method, at first, the specified number of cells (the optimum number of cells) is filled with reservoir oil and placed at reservoir temperature and at a pressure less than MMP pressure. Then as much as 1.2 volumes of pores were injected into them with gas. For each cell after gas injection, balance calculations are performed, and the amount of gas and liquid phases in each cell is calculated, and then the amount of extra injectable gas is transferred to the next cell. The amount of liquid and vapor phases in the last cell is calculated, and then by having the amount of x, y, the length of the Tie line in that pressure is calculated, and now the tie line length diagram is plotted according to the pressure, and then extrapolation is done to the point that the length of tie line is zero and that pressure will actually be the MMP.

## Method

In this study, the method of multiple mixing cells, including a series of fluid cells that are connected and initially filled with oil, has been used. Usually, the gas is mixed in the first cell with the test pressure and assuming complete mixing in the cell, the equilibrium phases are calculated. Then the extra volume of the cell (mostly balance gas) is transferred to the next cell and mixed with the liquid in the cell. This process continues for a series of cells until a certain volume of gas is injected; the primary multiple mixing cells methods are used to study the development of immiscibility rather than calculating the minimum immiscible pressure. In the following, the modeling method is discussed.

After calculating the compounds through the proximity of oil and gas, at this stage, the properties of the compounds and the desired pressure and temperature are initially investigated. In this paper, the cell-to-cell method is used to calculate MMP, so after entering the properties of the compounds, the number of cells should also be determined.

In the next step, the equilibrium ratio should be calculated first, and this ratio is the primary guess for calculating the compounds of each of the available phases. Wilson equation is used to calculate this ratio.

1 $$K_{i} = \left( {{\raise0.7ex\hbox{${P_{ci} }$} \!\mathord{\left/ {\vphantom {{P_{ci} } P}}\right.\kern-0pt} \!\lower0.7ex\hbox{$P$}}} \right)*\exp \left[ {5.37*\left( {1 + \omega_{i} } \right)*\left( {1 - \frac{{T_{ci} }}{T}} \right)} \right] \;\;\;for\;\;\; P < 3.5 {\text{Mpa}}$$

2 $$K_{i} = \left( {{\raise0.7ex\hbox{${P_{ci} }$} \!\mathord{\left/ {\vphantom {{P_{ci} } P}}\right.\kern-0pt} \!\lower0.7ex\hbox{$P$}}} \right)^{A - 1} *\left( {{\raise0.7ex\hbox{${P_{ci} }$} \!\mathord{\left/ {\vphantom {{P_{ci} } P}}\right.\kern-0pt} \!\lower0.7ex\hbox{$P$}}} \right)*\exp \left[ {5.37*\left( {1 + \omega_{i} } \right)*\left( {1 - \frac{{T_{ci} }}{T}} \right)} \right] \;\;\;for \,supercritical \,components$$

3 $$A = 1 - \left[ {\frac{{{\varvec{P}} - {\varvec{P}}_{{\varvec{a}}} }}{{{\varvec{P}}_{{\varvec{k}}} - {\varvec{P}}_{{\varvec{a}}} }}} \right]^{{\varvec{n}}}$$

After calculating the equilibrium ratios, the following equations are used to find the percentage of each component in the liquid and gas phases:

4 $${\varvec{x}}_{{\varvec{i}}} = \frac{{{\varvec{z}}_{{\varvec{i}}} }}{{1 + \left( {{\varvec{K}}_{{\varvec{i}}} - 1} \right){\varvec{n}}^{{\varvec{v}}} }}$$

5 $$y_{i} = \frac{{K_{i} z_{i} }}{{1 + \left( {K_{i} - 1} \right)n^{v} }}$$

Performing the above calculations requires finding the n^v^ that Rashford equations are used in this paper. Newton's method is used to solve these equations and obtain n^v^.

6 $$f\left( {n^{v} } \right) = \mathop \sum \limits_{i = 1}^{N} \frac{{z_{i} \left( {K_{i} - 1} \right)}}{{1 + \left( {K_{i} - 1} \right)n^{v} }}$$

With the help of initial guess n^v^ and Newton's method, the n^v^ value is obtained, and the calculations related to the compounds are performed through x_i_ and y_i_ equations. Then the state equilibrium equations are written for each liquid and gas phases. In this paper, PR equations are used. After solving the equations, and obtaining the roots of the equations, the fugacity and fugacity coefficient for each component are calculated using the Eq. (7)–(9).

7 $$ln\emptyset_{i} = \frac{{b_{i} }}{b}\left( {Z - 1} \right) - \ln \left( {Z - B} \right) - \frac{A}{{B( - 2\sqrt {2)} }}\left( {\frac{{2\mathop \sum \nolimits_{j = 1}^{N} x_{j} a_{ij} }}{a} - \frac{{b_{j} }}{b}} \right)ln\left( {\frac{{Z + \left( {1 - \sqrt 2 } \right)B}}{{Z + \left( {1 + \sqrt 2 } \right)B}}} \right)$$

8 $$a_{ij} = \left( {a_{i} a_{j} } \right)^{0.5} *\left( {1 - k_{ij} } \right)$$

9 $$fugacity \,coeficient:f_{i}^{phase} = \emptyset_{i}^{phase} *x \;or\; y_{i} *P$$

By calculating the coefficients of fugacity, the work reaches the error determination stage, and at this stage, by defining the following error, if the value of this error exceeds 10^–12^, the guess of the obtained k_i_ is not correct, and k_i_ must be corrected.

10 $$error = \sum \left( {1 - \frac{{f_{i}^{v} }}{{f_{i}^{l} }}} \right)^{2} < 1e - 12$$

In order to correct the ki values, the following formula has been used:

11 $$K_{i} = \frac{{f_{i}^{v} }}{{f_{i}^{l} }}$$

By performing sequential steps and reducing the error amount, the calculations have reached the final stage of the first cylinder. Now, with the help of x_i_ and y_i_ values, again by recombining the liquid and gas, a final compound is obtained through the following formula.

12 $$z_{i} = x_{i} + a*\left( {y_{i} - x_{i} } \right)$$

In this formula, a compound ratio is usually considered 0.5. After calculating the new compound, all the above steps will be done again to finish the number of cylinders considered in this article 30. After finishing the number of cylinders, the tie line value is calculated by combining the percentage of components in two gas and liquid phases through the following formula.To determine the number of cell cells in the existing algorithm, from 15 cells to 30, results were taken differently, and then for cells more than 30 others, there was negligible difference in the output data, and only the calculation time was long, so 30 cell cells were used in this study.

13 $$TL = \sqrt {\mathop \sum \limits_{i = 1}^{N} \left( {x_{i} - y_{i} } \right)^{2} }$$

**Figure 1:**
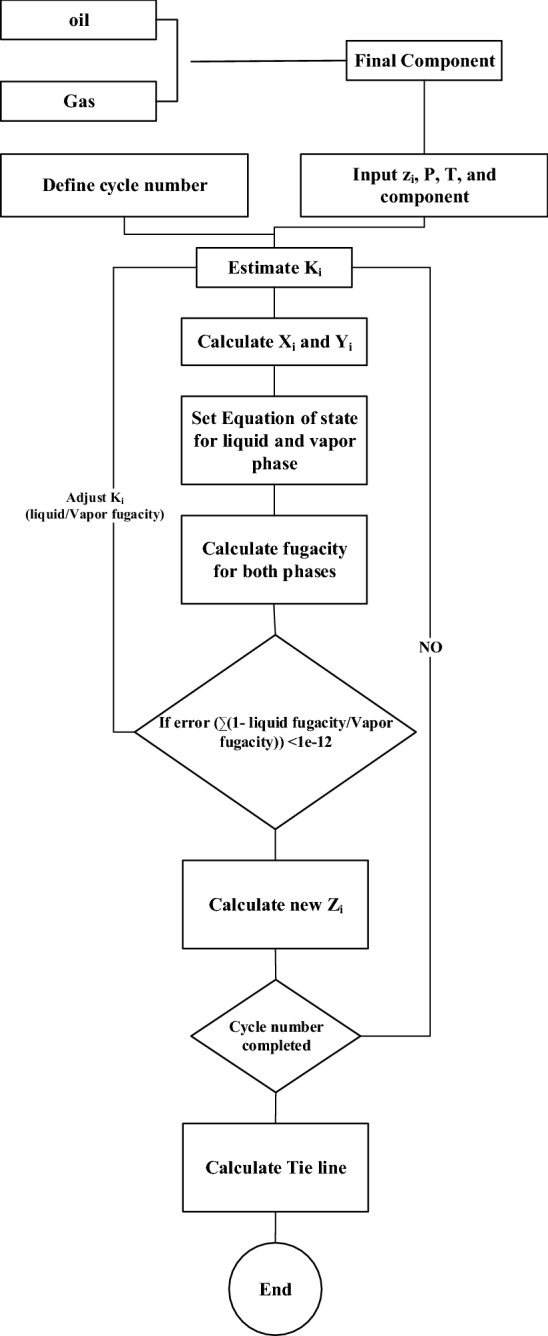
Algorithm for computing the CTC method.

The overall algorithm is shown by the flow chart in Fig. 1.

## Discussion

### Validation of model by comparing with experimental (laboratory) results

In order to evaluate the performance of the modeling performed using the mentioned algorithm in the method of doing the work, the results of the MMP obtained from the model have been evaluated and compared with the results of other sources.

**Figure 2:**
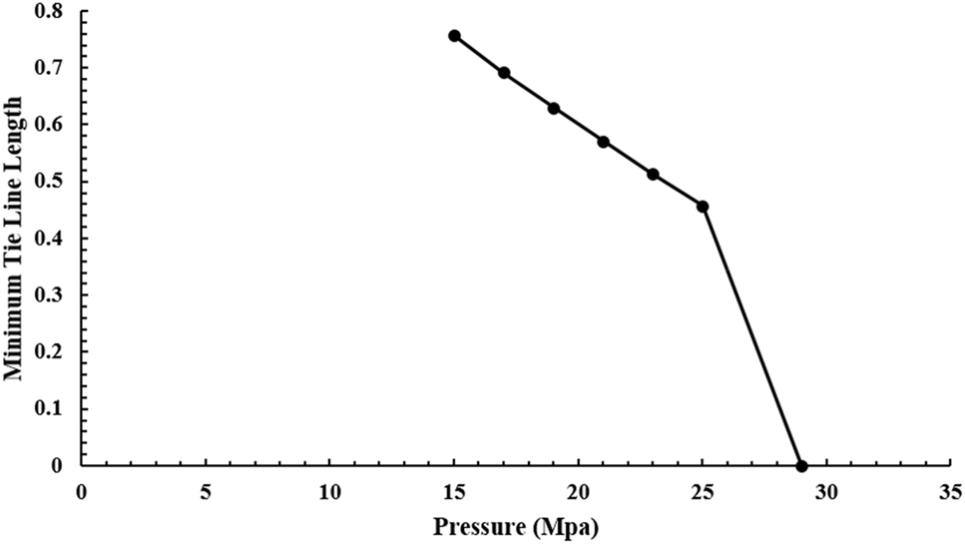
Tie line length diagram in terms of pressure when injecting methane into decane.

Figure 2 shows the length of the Tie Line in terms of pressure. According to the current theory, the pressure that Tie Line equals zero is equal to the MMP. As can be seen in Fig. 2, at a pressure of 29 MPa, this quantity is equal to zero and can indicate the MMP of methane gas injection to the normal decane. It should be noted that the modeling was performed at a temperature equal to the test temperature and modeling, and the ratio of compounds is the same as Mirzaei et al.^30^.

**Figure 3:**
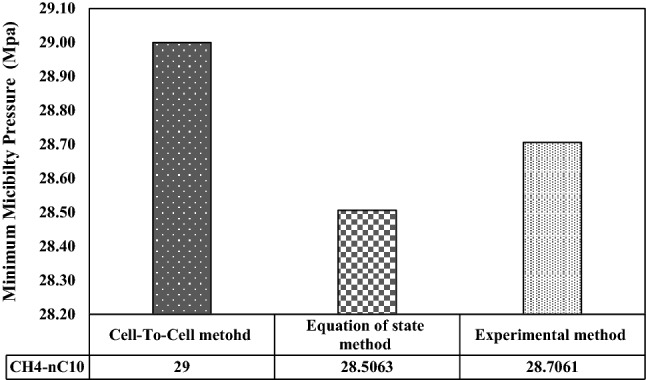
Comparison of MMP values calculated during methane injection into the decane (equally) from laboratory method, state equations, and CTC method.

Figure 3 is related to the amount of the MMP during the injection of methane gas into synthetic oil containing normal decane (a combination of 50% methane and 50% decane) and also compares it with experimental results and modeling in Mirzaei et al. studies^30^. The result obtained in this study is 1.731% using MMP calculation method versus the experimental results in Mirzaei paper and 1.023% error compared to the modeling results in Mirzaei paper, which can be concluded that the method used in this study has little error.

**Figure 4:**
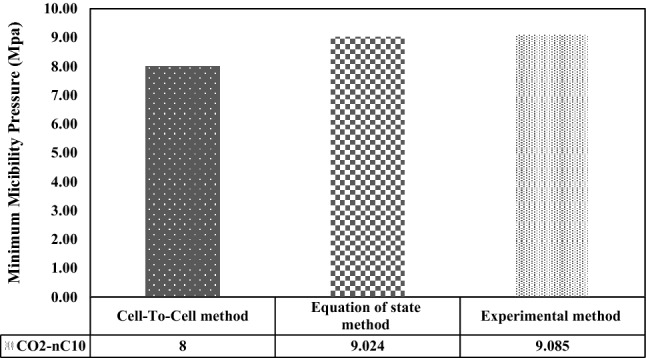
Comparison of MMP values calculated during carbon dioxide injection into the shop by laboratory method, state equations, and CTC method.

**Figure 5:**
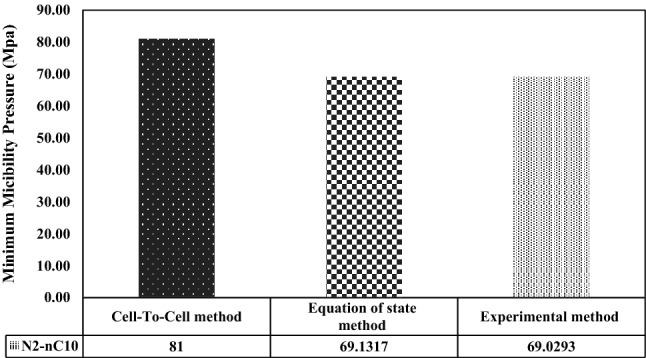
Comparison of MMP values calculated during nitrogen injection into the decane by laboratory method, state equations, and CTC method.

Also, a comparison of the MMP during the injection of other non-hydrocarbon gases was made. To compare the experimental (laboratory) data and modeling data in Mirzaei's paper with the results of modeling in this paper based on the tie-line length method for injection of N_2_ and CO_2_ gases into normal shops, Figs. 4 and 5 have been shown. It should be noted that this comparison has been made at the same temperature. This means that the modeling temperature in this study is equal to the modeling and laboratory temperatures related to Mirazi et al. paper.

As can be seen in Fig. 4, the MMP calculated in this modeling is 8 MPa, and the lowest MMP from modeling using the state equation in the method in Mirazi et al. paper is 9.02 MPa. The lowest amplified pressure obtained from the vanishing interfacial tension method mentioned in the article published by Mirzaei et al. is 9.09 MPa.

Figure 5 shows that the MMP calculated in this modeling is different from the results of the tests and modeling of Mirzaei et al. Therefore, these comparisons show that the modeling results for the injection of methane gas and hydrocarbons are more accurate.

### Comparison of three gases of methane, carbon dioxide, and nitrogen to normal-decane

**Figure 6:**
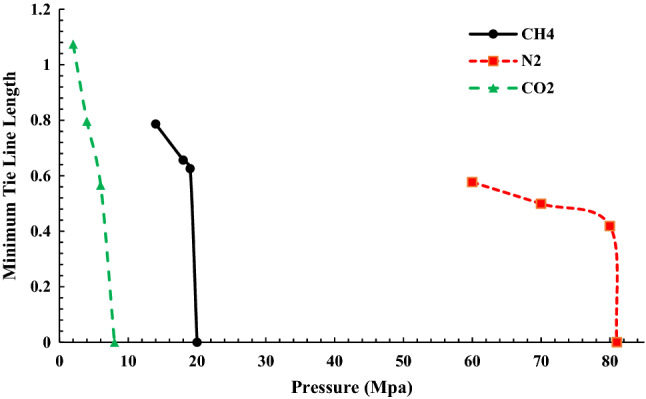
Comparison of MMP in the injection of methane, carbon dioxide, and nitrogen gases.

In this part of the study, the comparison of MMP in the injection of CO_2_, N_2_, and CH_4_ gases at 70 °C is compared, and the difference in MMP is shown in Fig. 6.

According to Fig. 6, as expected, due to the phase behavior of nitrogen, which tends to be more likely to be present in the gas phase at 70 °C, changing from gas phase to liquid phase requires very high pressure. Also, for carbon dioxide, due to its phase behavior, at 70 °C, it is more willing to be in the liquid state, so it achieves MMP with less pressure than nitrogen and methane. In other words, carbon dioxide's willingness for vaporising and condensing is more than other gases due to its phase behavior, which accelerates the miscibility process. As regards nitrogen, its phase behavior implies that nitrogen tends to vaporize instead of condensation, and miscibility occurs under high pressure. Moreover, due to the higher molecular weight of carbon dioxide comparing others, MMP for injecting CO_2_ into normal decane is less than other gases. The lowest MMP occurs for CO_2_ injection, which is 9 MPa; after that, MMP for methane is 20 MPa and 81 MPa for nitrogen. It is worth noting that in all three injections, the ratio of compounds is 0.7457 wt% of decane and 0.2543 wt% of injectable gas. The alteration in the slope of the tie-line can demonstrate the transmission from immiscible to the near-miscible stage and zero interfacial tension. Thus, this method can be used to predict near-miscible stages.

### Comparison of enriched-gas by NGL, LPG, and Naptha

The aforementioned results showed that the model could accurately measure MMP for hydrocarbon gases. The following is dedicated to enriching the injected gas by Naphta, LPG, and NGL, after which the rich gas is injected into the oil phase, and the MMP of the system is calculated and compared.

#### Injection of enriched gas by Naptha with different GOR

The percentage of injectable gas compounds for Naphta-enriched gas with GORs 1.25, 2.5, and 5 is in Table 1.

**Table 1:** Percentage of Naphta-enriched gas compounds with different GORs.

Component	GOR = 1.25 (Mscf/stb)	GOR = 2.5 (Mscf/stb)	GOR = 5 (Mscf/stb)
	Injection gas (1)	Injection gas (2)	Injection gas (3)
C1	53.03	69.31	81.87
C2	0.00	0.00	0.00
C3	0.00	0.00	0.00
i-C4	1.01	0.66	0.39
n-C4	0.05	0.03	0.02
I-C5	19.77	12.92	7.63
N-C5	6.25	4.08	2.41
C6	18.66	12.20	7.20
C7	1.22	0.80	0.47

**Figure 7:**
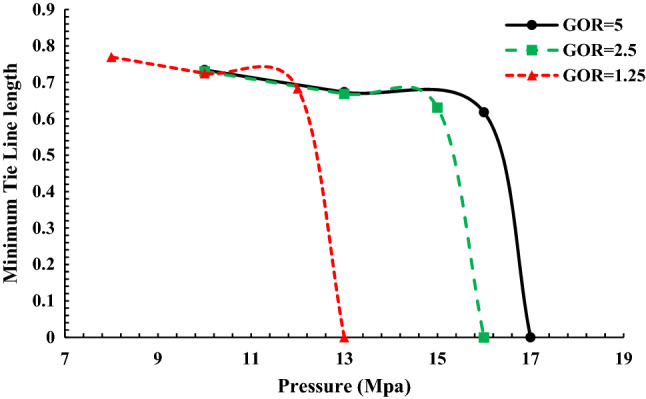
Tie line diagram when injecting Naphta-enriched gas into oil.

Figure 7 displays the length of the tie-line in terms of pressure to inject Naptha-enriched gas with a different GOR ratio of rich gas during injection into normal decane. The modeling was carried out at 70 °C.

According to Fig. 7, the MMP obtained in the injection of Naphta-enriched gas in different GOR changes from 13 to 17 MPa. As is evident from Fig. 7, with the increase of GOR, which means the reduction of heavy gas compounds, the MMP has shifted to higher values, resulting from an increase in the difference between oil and injectable gas in terms of heavy compounds and density with increasing GOR.

**Figure 8:**
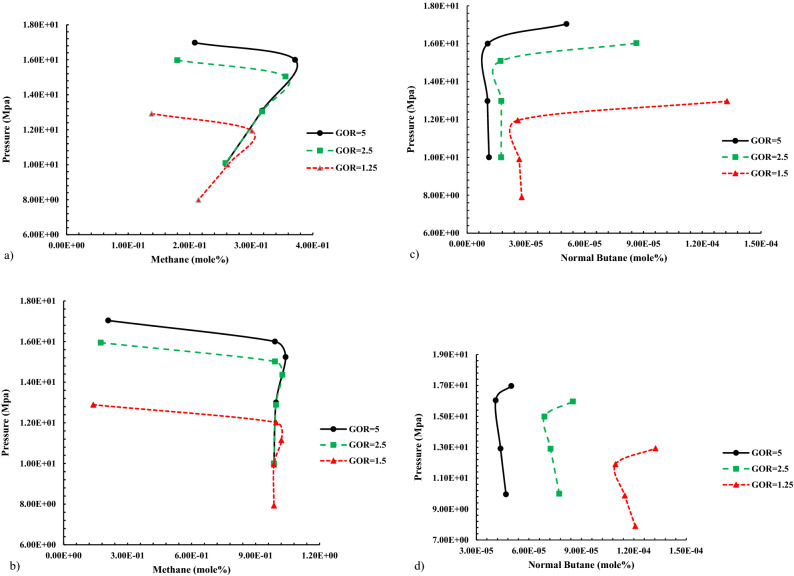
Altering compounds in the gas and liquid phases during gas injection enriched with various GOR by Naphta to normal decane. (**a**) Molar percentage C_1_ in liquid phase when injecting Naphta-enriched gas into C_10_. (**b**) Molar percentage C_1_ in gas phase when injecting Naphta-enriched gas into C_10_. (**c**) Molar percentage n-C_4_ in gas phase when injecting Naphta-enriched liquid into C_10_. (**d**) The molar percentage of n-C_4_ in the gas phase when injecting Naphta-enriched gas into C_10_.

According to Fig. 8, changes in the percentage of methane gas compounds (as a representative of dry and active gas in vaporizing mechanism) and normal butane (as a representative of rich and active compounds in condensing mechanism) in gas and liquid phases at pressures close to MMP.

By comparing the diagrams in Fig. 8, since the intensity of methane increase in the liquid phase was higher than the intensity of the reduction of the middle compounds; therefore, it can be considered that the dominant process in the above samples is the condensation of gas into the oil, which increases the amount of methane in the oil by increasing the pressure.

INotably, the slump in the percentage of methane in the liquid phase at near-miscible condition (near MMP and near-zero interfacial tension) implies that vaporizing mechanism help achieve a single-phase state at near miscibility condition.

#### Injection of enriched gas by LPG with different GOR

The percentage of injectable gas compounds (Table 2) and MMP diagram for LPG-enriched gas with GORs 1.25, 2.5, and 5 are as follows.

**Table 2:** Percentage of LPG-enriched gas compounds with different GORs.

Component	GOR = 1.25(Mscf/stb)	GOR = 2.5(Mscf/stb)	GOR = 5(Mscf/stb)
	Injection gas (1)	Injection gas (2)	Injection gas (3)
C1	94.56	94.78	94.89
C2	4.98	4.99	4.99
C3	0.22	0.11	0.05
n-C4	0.23	0.11	0.06
n-C5	0.01	0.01	0.01

**Figure 9:**
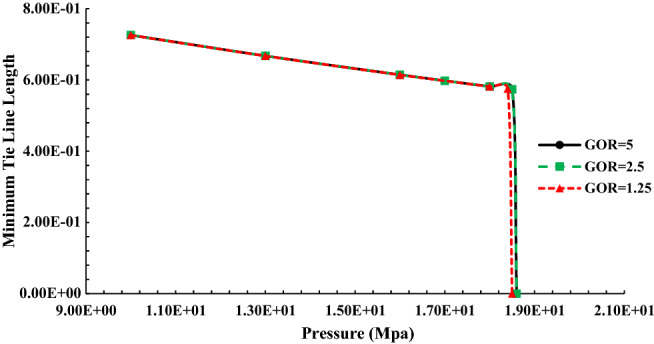
tie diagram line when injecting LPG-enriched gas into oil.

Figure 9 shows the length of the tie-line in terms of pressure to inject gas enriched by LPG with various gas-rich GORs to normal decane. Modeling is done at 70 °C.

According to Fig. 9, due to the insignificant differences between LPG-riched gases samples, there is a negligible difference between MMP. Similarly, by increasing GOR, the value of MMP is increased, which can be rooted in the oil and gas density difference.

According to Fig. 13, due to the lack of very low difference between light compounds of LPG samples, we see a very small difference between the MMP of different samples. In this diagram, with increasing GOR, we see an increase in the amphitheatric pressure caused by the difference in density between oil and gas.

**Figure 10:**
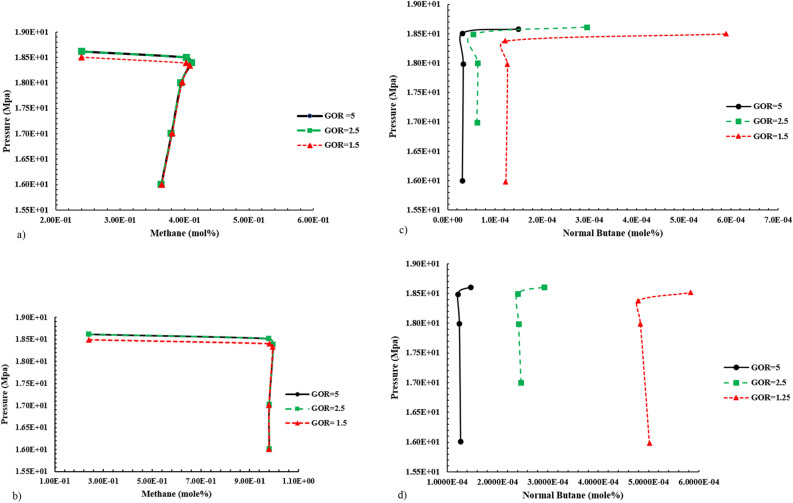
Altering compounds in the gas and liquid phase during gas injection enriched with various GOR by LPG to normal decane. (**a**) Molar percentage C_1_ in liquid phase when injecting gas enriched with LPG to C_10_. (**b**) Molar percentage C_1_ in gas phase when injecting gas enriched with LPG to C_10_. (**c**) Molar percentage n-C_4_ in liquid phase when injecting gas enriched with LPG to C_10_. (**d**) Molar percentage n-C_4_ in gas phase when injecting gas enriched with LPG to C_10_.

Figure 10 indicates the changes in the percentage of compounds in LPG-enriched gas at pressures close to MMP. According to the above diagrams, we see an increase in methane in the liquid phase with increasing pressure, which is more than the reduction of intermediate components in oil, which can be concluded that the dominant process is gas condensate into the oil. The reduction of methane concentration in the liquid phase is more than its reduction in LPG-enriched gas. To conclude, the amount of methane drained from the liquid phase and reached to initial thermodynamic condition.

#### Injection of enriched gas by NGL with different GOR

The percentage of injectable gas compounds and MMP diagram for NGL-enriched gas with GORs 1.25, 2.5, and 5 are as follows (Table 3).

**Table 3:** Percentage of NGL-enriched gas compounds with different GORs.

Component	GOR = 1.25(Mscf/stb)	GOR = 2.5(Mscf/stb)	GOR = 5(Mscf/stb)
	Injection gas (1)	Injection gas (2)	Injection gas (3)
C1	94.5661	94.7825	94.8912
C2	5.0950	5.0476	5.0238
C3	0.1611	0.0808	0.0404
i-C4	0.0343	0.0172	0.0086
n-C4	0.0740	0.0371	0.0186
I-C5	0.0209	0.0105	0.0052
N-C5	0.0220	0.0110	0.0055
C6	0.0148	0.0074	0.0037
C7	0.0051	0.0026	0.0013
C8	0.0033	0.0016	0.0009
C9	0.0022	0.0011	0.0005
C10	0.0012	0.0006	0.0003

**Figure 11:**
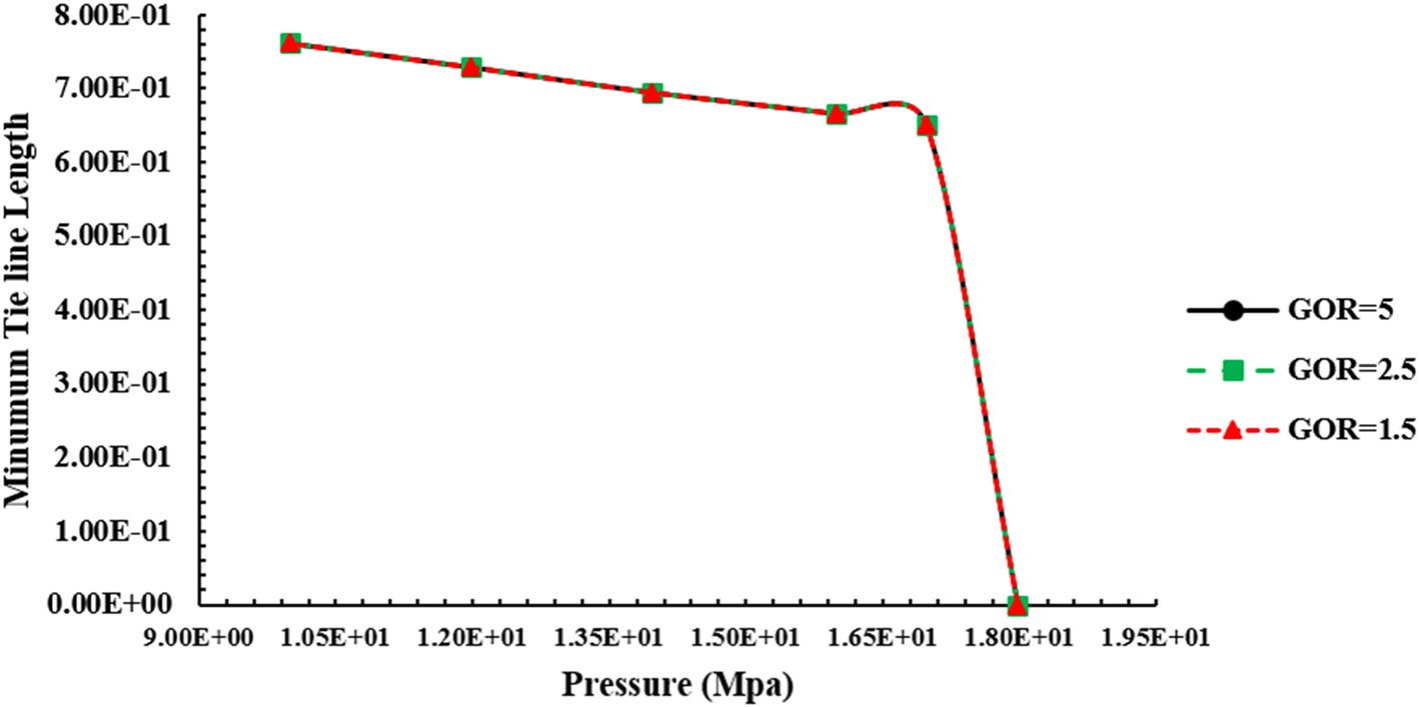
Tie diagram line when injecting NGL-enriched gas into oil.

Figure 11 illustrates the tie line length versus pressure for injecting NGL-enriched gas with different GOR to normal decane. According to Fig. 11, due to the negligible differences among all sample compositions, there is small difference among MMP of samples. Similarly, by decreasing GOR, miscibility occurs in lower pressure.

**Figure 12:**
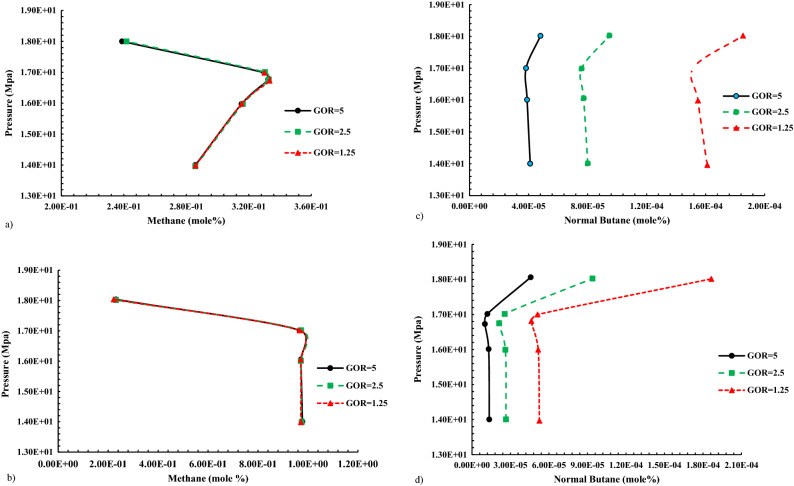
Altering compounds in gas and liquid phases during gas injection enriched with different GOR by NGL to normal decane. (**a**) The molar percentage C_1_ in liquid phase when injecting gas enriched with NGL to C_10_. (**b**) The molar percentage C_1_ in gas phase when injecting gas enriched with NGL to C_10_. (**c**) The molar percentage n-C_4_ in liquid phase when injecting NGL-enriched gas into C_10_. (**d**) The molar percentage of n-C_4_ in gas phase when injecting NGL-enriched gas into C_10_.

Changes in the percentage of compounds in NGL-enriched gas at pressures close to MMP can be witnessed in Fig. 12.

According to the above diagrams, the increase in the molar percentage of methane in the liquid phase by increasing the pressure is more than the reduction of the middle components in the liquid phase, which can indicate that the dominant process in NGL injection is condensation. In addition, it can be observed that the methane concentration decreases significantly in the liquid phase.

#### Comparison between different gases

**Figure 13:**
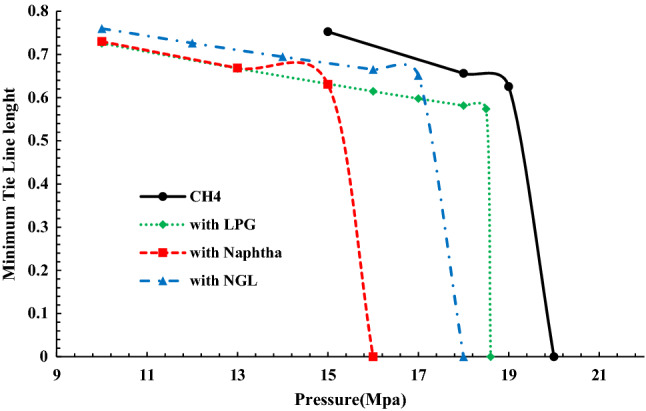
Comparison of the tie-line length diagram in terms of pressure for CH_4_ gas alone and CH_4_ gas enriched with NGL, LPG, Naphta.

In this section, a comparison between the injection of different gases, including pure Ch_4_ and enriched gases with 2.5 GOR (which is used in the industry) has been made (Fig. 13).

According to the table of compounds previously introduced, the amount of heavy compounds of Naphta-enriched gas is higher than the other two and also has heavier compounds close to oil in terms of density than pure methane, so it was expected that in enriched-gas with Naphta we would see the MMP that confirms the simulation results. Also, because methane has the highest difference from oil in density and compounds, we need higher pressure than other gases to achieve miscibility pressure.

Therefore, the enrichment of gas reduces the MMP and consequently reduces economic costs in gas injection projects.

## Conclusion

According to the simulations and comparison with the previous laboratory and theory methods, it was found that the method presented by the algorithm presented in this paper is accurate for calculating the MMP. According to the modeling, the following results can be noted:

- This algorithm will be able to determine the conditions of near-miscible injection by calculating the slope of the Tie line length diagram in terms of pressure. Therefore, one of the advantages of this proposed method is determining the miscibility, near-miscibility, and immiscible conditions. Injection in near-miscible conditions to some oil reservoirs (conventional reservoirs) has more recovery than miscible and immiscible injection.
- TUsing rich gas reduces the miscible pressure of oil and gas to 16 MPa. This amount is 20 MPa if methane is used, so enrichment of gas is a suitable method to reduce the pressure needed to inject gas into oil tanks, which is very effective in reducing the economic costs of the project. The reason for reducing the miscible pressure is the approach of the gas composition to the reservoir oil and the presence of heavier compounds in rich gas than in dry gas.
- Methane percentage as a light compound and percentage of intermediate compounds in liquid and gas phases in each cell helps to identify the governing mechanism in each compressive interval and in the transfer from each molecule to another cell. Therefore, one of the advantages of using this proposed algorithm for determining MMP is identifying the mechanism governing different thermodynamic conditions.
- The usage of Naptha to increase intermediate components of dry gas performs better than LPG and NGL, leading to the lowest MMP.
- Condensing mechanism is the most important mechanism in the use of rich compounds during gas injection, which reduces the MMP compared to the use of dry gas. In this mechanism, gas compounds enter the oil.
- By increasing the ratio of dry gas to rich gas (GOR) from 1.25 to 5 Mscf/Stb, the MMP increases in the presence of all three types of rich gas.
- Comparing the injection of three gases of nitrogen, carbon dioxide, and methane into the normal shop or using the algorithm presented in this paper, it can be observed that the use of carbon dioxide will have the lowest MMP. Also, due to the environmental damage of this gas in case of presence in the air, it is important to use this gas to increase the recovery of oil reservoirs and prevent environmental damage.

According to the simulations and comparison with the previous laboratory and theory methods, it was found that the theory method of multiple mixing cells has good accuracy in calculating the MMP. As the results showed, the use of rich gas reduced the MMP from 20 to 16 MPa, so enrichment of gas as a suitable method to reduce the pressure required to inject gas into the oil reservoirs is very effective in reducing the economic costs of the project.

## Data Availability

All data generated or analysed during this study are included in this published article.

## References

[1] Kumar J, Agrawal P, Draoui E (2017). A case study on miscible and immiscible gas-injection pilots in a middle east carbonate reservoir in an offshore environment. SPE Reserv. Eval. Eng..

[2] Rommerskirchen, R., Nijssen, P., Bilgili, H., & Sottmann, T. Reducing the miscibility pressure in gas injection oil recovery processes. In *Abu Dhabi International Petroleum Exhibition and Conference*. OnePetro 10.2118/183389-MS (2016).

[3] Ahdaya, M., Imqam, A. Miscible gas injection application for enhanced oil recovery: Data analysis. In *54th U.S. Rock Mechanics/Geomechanics Symposium*. p. ARMA-2020–1035, (2020).

[4] Welge HJ, Johnson EF, Ewing SP, Brinkman FH (1961). The linear displacement of oil from porous media by enriched gas. J. Pet. Technol..

[5] Wachmann C (1964). A mathematical theory for the displacement of oil and water by alcohol. Soc. Pet. Eng. J..

[6] Zhao H, Fang Z (2020). Improved multiple-mixing-cell method for accelerating minimum miscibility pressure calculations. SPE J..

[7] Dindoruk B, Johns R, Orr FM (2021). Measurement and modeling of minimum miscibility pressure: a state-of-the-art review. SPE Reserv. Eval. Eng..

[8] Sinha U, Dindoruk B, Soliman M (2021). Prediction of CO_2_ minimum miscibility pressure using an augmented machine-learning-based model. SPE J..

[9] Wan, T., Meng, X., Sheng, J. J., Watson, M. Compositional modeling of EOR process in stimulated shale oil reservoirs by cyclic gas injection. In *SPE Improved Oil Recovery Symposium*. OnePetro, 10.2118/169069-MS (2014).

[10] Emera MK, Sarma HK (2005). Use of genetic algorithm to predict minimum miscibility pressure (MMP) between flue gases and oil in design of flue gas injection project. Renew. Energy.

[11] Saini, D., Rao, D. N. Experimental determination of minimum miscibility pressure (MMP) by gas/oil IFT measurements for a gas injection EOR project. In *SPE Western Regional Meeting*. OnePetro. 10.2118/132389-MS (2010).

[12] Chen, H. *et al*. Empirical correlation of minimum miscible pressure of pure and impure CO_2_ flooding. In *Carbon Management Technology Conference*. OnePetro 10.7122/CMTC-553599-MS (2019).

[13] Alshuaibi, M., Farzaneh, S. A., Sohrabi, M., Mogensen, K. An accurate and reliable correlation to determine CO_2_/crude oil MMP for high-temperature reservoirs in Abu Dhabi. In *Abu Dhabi International Petroleum Exhibition and Conference*. OnePetro, 10.2118/197344-MS (2019).

[14] Emera, M. K., Lu, J. Genetic algorithm (GA)-based correlations offer more reliable prediction of minimum miscibility pressures (MMP) between the reservoir oil and CO_2_ or flue gas. In *Canadian International Petroleum Conference*. OnePetro 10.2118/2005-003 (2005).

[15] Jessen K, Stenby EH, Orr FM (2004). Interplay of phase behavior and numerical dispersion in finite-difference compositional simulation. SPE J..

[16] Kanatbayev, M., Meisingset, K. K., Uleberg, K., Asa, S. SPE-173827-MS comparison of mmp estimation methods with proposed workflow, [Online]. Available: http://onepetro.org/SPEBERG/proceedings-pdf/15BERG/All-15BERG/SPE-173827-MS/1405953/spe-173827-ms.pdf/1 (2015).

[17] Johns RTT, Ahmadi K, Zhou D, Yan M (2010). A practical method for minimum-miscibility-pressure estimation of contaminated CO_2_ mixtures. SPE Reserv. Eval. Eng..

[18] Ungar F, Ahitan S, Worthing S, Abedini A, Uleberg K, Yang T (2022). A new fluidics method to determine minimum miscibility pressure. J. Pet. Sci. Eng..

[19] Zhou Y, Wu X, Zhong X, Zhang S, Pu H, Zhao JX (2020). Development of silicon quantum dots based nano-fluid for enhanced oil recovery in tight Bakken cores. Fuel.

[20] Ahmadi MA, Zendehboudi S, James LA (2017). A reliable strategy to calculate minimum miscibility pressure of CO_2_-oil system in miscible gas flooding processes. Fuel.

[21] Li R, Li H (2019). A Modified multiple-mixing-cell algorithm for minimum miscibility pressure prediction with the consideration of the asphaltene-precipitation effect. Ind. Eng. Chem. Res..

[22] Hutchinson CA, Braun PH (1961). Phase relations of miscible displacement in oil recovery. AIChE J..

[23] Jensen, F., Michelsen, M. L. Calculation of first contract and multiple contact minimum miscibility pressures, In Situ;(USA) **14**(1) ISSN 0019-3267, (1990).

[24] Orr, F. M. *Theory of Gas Injection Processes* (2005).

[25] McGuire PL, Okuno R, Gould TL, Lake LW (2017). Ethane-based enhanced oil recovery: an innovative and profitable enhanced-oil-recovery opportunity for a low-price environment. SPE Reserv. Eval. Eng..

[26] AltOn B (1969). Realistic K Values of C,+ hydrocarbons for calculating oil vaporization during gas cycling at high pressures. J. Pet. Technol..

[27] Jaubert JN, Wolff L, Neau E, Avaullee L (1998). A very simple multiple mixing cell calculation to compute the minimum miscibility pressure whatever the displacement mechanism. Ind. Eng. Chem. Res..

[28] Metcalfe RS, Fussell DD, Shelton JL (1973). A multicell equilibrium separation model for the study of multiple contact miscibility in rich-gas drives. Soc. Pet. Eng. J..

[29] Kariman Moghaddam A, Saeedi Dehaghani AH (2017). Modeling of asphaltene precipitation in calculation of minimum miscibility pressure. Ind. Eng. Chem. Res..

[30] Mirzaie M, Tatar A (2020). Modeling of interfacial tension in binary mixtures of CH_4_, CO_2_, and N_2_ - alkanes using gene expression programming and equation of state. J. Mol. Liq..

